# Establishing healthy longevity clinics in publicly funded hospitals

**DOI:** 10.1007/s11357-024-01132-0

**Published:** 2024-03-21

**Authors:** Sara L. R. Bonnes, Tzipora Strauss, Allyson K. Palmer, Ryan T. Hurt, Louis Island, Abigail Goshen, Laureen Y. T. Wang, James L. Kirkland, Evelyne Bischof, Andrea B. Maier

**Affiliations:** 1 Department of Medicine, Mayo Clinic, Rochester, MN USA; 2 Sheba Longevity Center, Sheba Medical Center, Tel Hashomer, Israel; 3 Tel Aviv Faculty of Medicine, Tel Aviv University, Tel Aviv, Israel; 4 Healthy Longevity Translational Research Programme, Yong Loo Lin School of Medicine, National University of Singapore (NUS), Singapore, 117456 Singapore; 5 Centre for Healthy Longevity, @AgeSingapore, National University Health System (NUHS), Singapore, Singapore; 6 Department of Epidemiology and Preventive Medicine, School of Public Health, Faculty of Medicine, Tel Aviv University, Tel Aviv, Israel; 7 Well Programme, Alexandra Hospital, National University Health System (NUHS), Singapore, Singapore; 8 Renji Hospital of the Jiaotong University School of Medicine, Department of Oncology and Clinical Cancer Center, State Key Laboratory of Oncogenes and Related Genes, Shanghai, China; 9 Department of Human Movement Sciences, @AgeAmsterdam, Faculty of Behavioural and Movement Sciences, Vrije Universiteit Amsterdam, Amsterdam Movement Sciences, Amsterdam, Netherlands

**Keywords:** Publicly funded hospitals, Healthy longevity clinics, Academic healthy longevity

## Abstract

Healthy longevity medicine integrates geroscience and other disciplines into clinical settings, aiming to optimize health throughout one’s lifespan. Multiple factors have led to increased consumer engagement, with private clinics currently meeting the demand for guidance to improve healthy longevity. The establishment of healthy longevity clinics in publicly funded hospitals is a significant development, making longevity-focused healthcare more accessible. These clinics rely on multidisciplinary teams of physicians and allied health professionals. Diagnostics involve comprehensive evaluations of medical history, physical examinations, and various clinical tests to detect early signs of age-related functional decline. Interventions in healthy longevity medicine encompass lifestyle modifications, supplements, repurposed drugs, and social and environmental interventions. Collaboration with research institutions and industry partners is crucial for advancing healthy longevity medicine and creating standardized protocols. In this article, we review the process of creating healthy longevity clinics in public hospitals to ensure the best possible care for individuals pursuing healthy longevity.

## Introduction

Life expectancy has risen in the past century due to advancements in disease prevention and treatment. According to the World Health Organization (WHO), the global population of individuals aged 60 years and above is projected to rise from 12 to 22% between 2015 and 2050 [1]. With this shift in demographics, diseases associated with older age are becoming more prevalent [2]. In 2016, industrialized countries reported an average of approximately 11 years living with chronic disease, significantly impacting individuals’ quality of life, placing substantial demands on healthcare systems, and presenting economic challenges for societies.

Driven by the need to address the impact of aging, there is a growing focus on geroscience, the study of biological processes of aging and their relationship to age-related dysfunction and disease. Healthy longevity medicine, which integrates geroscience and other disciplines into clinical settings, is gaining momentum. Healthy longevity medicine aims to optimize health and healthspan by targeting aging processes throughout one’s lifespan. The growing understanding of how to measure and address biological ageing processes has led to increasing consumer engagement, and private clinics have emerged to cater to the growing demand from individuals seeking guidance on diagnostics and interventions to improve their healthy longevity.

An important development is now taking place as the first healthy longevity clinics in publicly funded hospitals are being established. Hospitals integrating the latest evidence-based healthy longevity medicine approaches into their practices mark a significant step forward in making longevity-focused healthcare accessible to a wider population. This approach will help develop standards of care for healthy longevity clinics and moving research forward. This article will outline the process that these academic centers have pursued to establish their healthy longevity clinics and discuss best practices as other institutions also develop healthy longevity clinics.

## Forming an international collaboration

In response to the aging populations across the globe, patient inquiry, and a desire to advance the science of healthy longevity medicine, National University Health System, Sheba Medical Institute, and Mayo Clinic all independently started to develop healthy longevity clinics. Their independent efforts were united through collaborative networking of the Healthy Longevity Medical Society.

While the institutions are quite distinct in background and location, there are common threads that were drivers for each of these centers to start an academic healthy longevity clinic. See the table below for information about each of the institutions.

**Table:** 

Institution	Location	Facility size	Clinic launch	Services available
Mayo Clinic	Rochester, MN, USA	Approximately 2000 bed teaching hospital	July 2023	Body composition, physical performance testing, physician assessment, dietitian, exercise specialist, physical therapist, mind body counselors, health and wellness coaching, research partnership with the NIH Translational Geroscience Network, and its Facility for Geroscience Analysis as well as the Robert and Arlene Kogod Center on Aging and others. Generally seeing individuals greater than age 40.
National University Health System - Alexandra Hospital	Singapore	Network of teaching hospitals with 2265 beds	August 2023	Clinical and biological assessment followed by lifestyle and medical interventions provided by a multidisciplinary team. Inclusion of healthy individuals aged 35 years and older or individuals with a stable chronic disease aged 35–70 years. The clinic is a partnership with National University of Singapore’s School of Medicine and National University Health System.
Sheba Medical Center Tel - Hashomer	Israel	1900 bed university and teaching hospital	November 2023	Comprehensive assessment of multiple systems—cardiovascular, sensory, neuropsychiatry, cognitive, stamina and optimal individual performance, as well as biological age and hallmarks of aging. Personalized in-house app-based guided interventions with ongoing monitoring. Includes individuals from 45 years on.

## Patient inquiry

Health issues have become prevalent, and many individuals seek knowledge how to prevent age-related diseases and how to improve quality of life. Given the wealth of information and differing recommendations on how to achieve optimal health, individuals have been asking for evidence-based recommendations from hospitals and academic institutions.

## Advancing the science

Geroscience and translational geroscience research has been growing. With the centralized expertise of multiple specialities and research teams, academic centers are poised to advance the science from preclinical research based on animal studies, to human diagnostic and intervention studies and integrate this work into healthy longevity clinics. With international collaboration and patient populations that are geographically and ethnically diverse, we hope to allow for more robust clinical trials.

## Accessible healthcare

Much of the global population is not able to afford care at private healthy longevity clinics. Developing clinics at academic centers improves accessibility of healthy longevity medicine.

## Integration with existing healthcare clinics

While optimizing health and aiming for the absence of pathological conditions is a primary objective of healthy longevity medicine, it is important to acknowledge that most individuals are likely to experience one or more health issues at some point in their lives. To facilitate seamless and efficient transitions in patient care, healthy longevity clinics should maintain close collaboration with primary care and conventional medical specialties. This collaborative approach ensures that when the focus needs to shift towards symptom evaluation and treatment of a specific disease, the transition can be accomplished efficiently, effectively, and through collaborative approaches that continue to incorporate an individuals longevity goals.

## Challenges in establishing a healthy longevity clinic

Creating a healthy longevity clinic within a publicly funded hospital poses many challenges, including but not limited to funding the service, identifying a minimum data set of health assessments and outcomes, keeping pace with technological innovation, balancing commercial interests with evidence-based medicine, and ensuring electronic medical record (EMR) interoperability.

Despite the increasing demand for personalized, proactive healthcare, few resources are spent on preventative measures. While private clinics have been able to utilize direct to consumer charging, at publicly funded hospitals, codes for billing insurance have to be identified or other models have to be introduced to reduce costs charged to individuals directly. In addition, due to institutional regulatory limitations, each of these clinics has found that testing, documentation, and treatment options are limited by institutional policy. Many diagnostic tests available in private healthy longevity clinics have not yet met the criteria required for use at our institutions. Even if they do meet these criteria, some of the reporting mechanisms cannot be incorporated into the EMR or do not meet the encryption/ data safety requirements to allow direct sharing with the institution. Given the challenges faced so far, it is anticipated that incorporation of new tests and interventions will be delayed as these will need to be thoroughly vetted by institutional committees and assessed for data protection.

## The clinical team

**Fig. 1:**
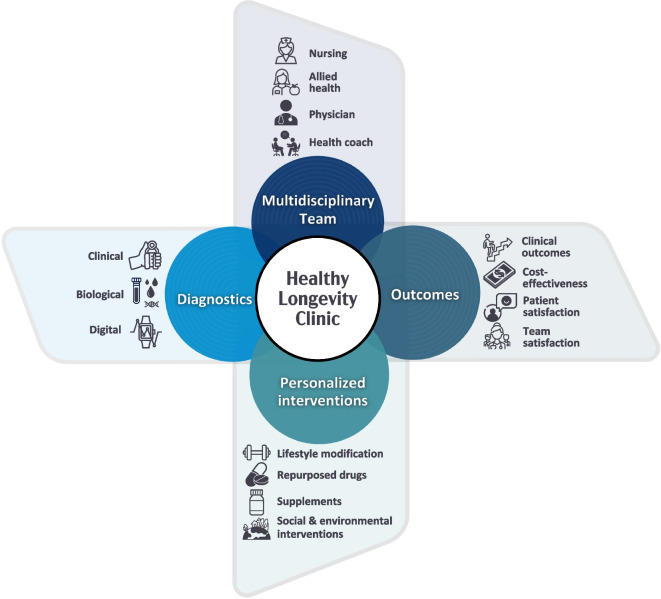
Representation of the multidisciplinary team and diagnostic and intervention approaches in academic healthy longevity clinics

Many team members are involved in a healthy longevity clinic in a publicly funded hospital. See Fig. 1 for a graphic representation of this collaboration.

Healthy longevity medicine physicians have expertise in various fields and a comprehensive understanding of the underlying biological processes of aging, such as proteostasis, epigenetics, and cellular senescence, which are associated with age-related chronic diseases [3]. Nurses help with the intake process, ensure accuracy of patient medical information, and measure biomarkers of ageing [4]. Nurses also play a crucial role in individual education and follow up.

Allied health professionals such as dietitians provide personalized dietary plans promoting optimal nutrition for healthy longevity. Physiotherapists and exercise physiologists deliver tailored exercise programs to improve mobility, muscle strength, and overall physical well-being. Mental health professionals address the emotional, motivational, and cognitive aspects of aging. Pharmacists may review medication and supplement lists for accuracy and help avoid drug-drug or drug-supplement interactions. Health coaches focus on promoting healthy lifestyle and overall guidance by emphasizing behavior changes and empowerment.

## Healthy longevity diagnostics

Diagnostics provide essential insights into the status of the aging process including the rate at which age-related changes occur and the overall health status of organ systems. Healthy longevity diagnostics (“gerodiagnostics”) are based on biomarkers of aging [4] including biological, clinical, and digital diagnostics.

While the offerings in each of the clinics varies, the following are key components to include in a healthy longevity assessment. Clinical diagnostics encompass a thorough assessment of an individual’s medical history, physical exam, and various tests, including cognitive, mental, and social interaction evaluations, along with assessments of sleep quality, architecture, and nutritional status and intake. Additionally, imaging techniques such as body composition and bone densitometry are employed. Physical performance is gauged through the 6-min walk test, muscle strength measurement, and cardiopulmonary exercise testing among others. However, the objective of this testing is not to identify existing pathologies, but rather to uncover early indicators of age-related functional decline and to create targeted interventions to slow or reverse it [5]. Utilizing responsive clinical gerodiagnostics, healthcare practitioners can also track intervention effectiveness and make informed decisions regarding ongoing care [4].

Molecular and cellular biomarkers may involve the measurement of basic laboratory parameters, including complete blood count, liver and renal function, lipid, inflammatory, and metabolic profiles. They may extend to an analysis of the genome, epigenome, transcriptome, proteome, metabolome, and microbiome, as well as specific molecules, cellular structures, or cell types. By assessing molecular and cellular markers, healthcare professionals may gain valuable information about the individual’s biological age, ability to respond to such stresses as infection or trauma, identify potential health risks, and tailor interventions to optimize health.

Digital diagnostics leverage technological advancements to gather and analyze physiological systems and lifestyle data. These may involve wearable devices, smartphone applications, and other digital platforms that track and monitor physical activity, sleep patterns, nutrition, heart rate variability, temperature, hearing, visual tracking, breathing rate, or glucose levels. Evidence-based protocols have yet to be established for utilization of such data, frequency of assessments, and incorporation into the EMR. Leveraging various methods such as artificial intelligence (AI) and machine learning may improve the understanding of individual biological aging, identify novel gerodiagnostics, and help to tailor personalized recommendations for health optimization.

## Interventions

While there would be greater simplicity in an algorithm-based approach to interventions in a healthy longevity clinic, we as an international team continue to assess how to incorporate the unique needs of each individual and their goals into prescribed interventions. There are four key components to interventions recommended from a healthy longevity clinic: (1) lifestyle interventions, (2) repurposed drugs, (3) supplements, and (4) social and environmental interventions.

1. Lifestyle interventions
  The six key pillars of a healthy lifestyle are (1) healthful eating, (2) physical activity, (3) managing stress, (4) restorative sleep, (5) social connections, and (6) avoiding use of risky substances (generally considered as tobacco, illicit drugs, misuse of prescription drugs and alcohol). Given the strong evidence to support lifestyle interventions, these are a key foundation to every individual . While some individuals may excel in all these domains, others seek consultation for a specific aspect of lifestyle, such as how to improve diet, or what type of exercise is best for them.

2) Repurposed drugs
  Repurposing drugs involves utilizing existing medications for new therapeutic purposes. In the context of healthy longevity medicine, researchers are investigating the potential of certain drugs approved for other conditions to modify the aging process or delay the onset of age-related diseases [6]. Examples of repurposed drugs studied in healthy longevity medicine include metformin and SGLT2 inhibitors, commonly used for diabetes management [6], and rapamycin, originally developed as an immunosuppressant [7]. These drugs have shown promising effects in preclinical and clinical studies, and further research is needed to understand their potential benefits and risks in optimizing health.

3) Supplements
  Supplements are often a part of healthy longevity medicine care plans. These include compounds showing promise in optimizing lifespan in animal models, but often also enhance performance or alleviate dysfunction in early clinical studies in humans [8]. Among these compounds, nicotinamide mononucleotide (NMN) [9], alpha-ketoglutarate (AKG) [10], glycine [11], senolytics including fisetin and the combination of dasatinib plus quercetin (D + Q) [12], urolithin A [13], and spermidine [14] have garnered attention for their potential geroprotective effects. Additional clinical trials will be crucial in firmly establishing the potential role of supplements in delaying, preventing, alleviating, or treating diseases and disorders linked to fundamental aging mechanisms.

4) Social and environmental interventions
  Social and environmental interventions are crucial in optimizing health by improving physical function, cognitive performance and quality of life. Social interventions encompass various strategies including community engagement programs, social support networks, and educational initiatives to enhance cognitive and physical abilities.

## Putting it all together

**Fig. 2:**
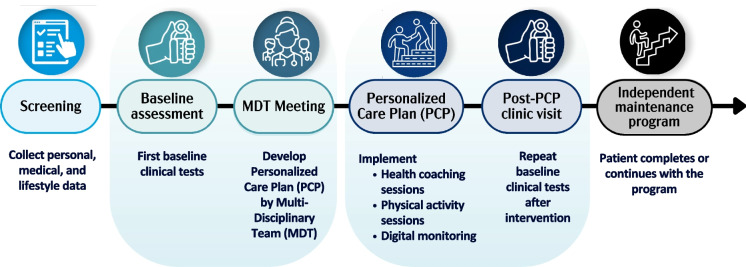
Participant journey during healthy longevity medicine care delivery

While the flow at each institution varies, Fig. 2 provides a general representation of the patient experience through each clinic.

## Assessment of viability

Because private clinics operate on a for-profit basis, assessment of their ongoing viability will generally operate almost exclusively on a finance-driven basis. The decision to continue operations of a healthy longevity clinic in a publicly funded hospital has multiple factors in addition to financial performance to consider. While the metrics to assess ongoing support of clinical areas is different in each hospital, some considerations generally include (1) financial performance, including grant funding for research in this domain; (2) patient satisfaction; and (3) patient reported outcomes including quality of life/improved health metrics. Opportunities to participate in clinical trials and advancing the science by international collaboration with other academic healthy longevity clinics will likely contribute to growth of similar clinics in other hospitals, and additional academic collaboration.
